# Roles of the Complement System in Peripheral Nerve Injury and Repair

**DOI:** 10.3390/bioengineering13020251

**Published:** 2026-02-20

**Authors:** Carmelina Azar, Kaixin Pan, Prini Jain, Elsa Sanchez-Lopez, Sameer B. Shah

**Affiliations:** 1 Department of Biological Sciences, University of California San Diego, La Jolla, CA 92093, USA; cazar@hs.uci.edu (C.A.); p7jain@ucsd.edu (P.J.); 2 Department of Bioengineering, University of California San Diego, La Jolla, CA 92093, USA; k1pan@ucsd.edu; 3 Department of Orthopaedic Surgery, University of California San Diego, La Jolla, CA 92093, USA; esl023@health.ucsd.edu; 4 Research Service, VA San Diego Healthcare System, San Diego, CA 92161, USA

**Keywords:** peripheral nerve injury, complement, immune system, inflammatory, immune cell, nerve regeneration, neuron, Wallerian degeneration

## Abstract

When nerves are severed, such as during traumatic injury, an acute injury state is induced, characterized by biological and physical changes in the proximal and distal stumps. Beyond the initial injury phase, over a time frame of weeks to months, nerves that remain unrepaired progressively enter a chronic injury state, characterized by a change in the extracellular matrix structure of the distal stump, the down-regulation of neurotrophic factors and the loss of macrophages’ and Schwann cells’ ability to clear out degraded axons and myelin. There are also potential systemic impacts away from the site of injury, including in end organs such as muscle and bone. The literature suggests that several of these processes may be strongly influenced by innate and adaptive immune system responses, including a major role for complement pathways. This review details evidence in favor of such a possibility, as well as knowledge gaps and areas for future investigation.

## 1. Acute and Chronic Phases of Traumatic Nerve Injury in the PNS

The peripheral nervous system (PNS) is composed of nerves branching out from the spinal cord, allowing for widespread communication between the central nervous system and the body. Nerves are a composite tissue consisting of many cell types [1]. The primary electrical cells of peripheral nerves—neurons—are ensheathed by myelinating or non-myelinating Schwann cells and are surrounded by endoneurial connective tissue. Many nerve fibers bundled together form a fascicle, which is surrounded by the perineurium, a second connective tissue layer. A typical nerve is composed of several fascicles, which are surrounded by the epineurium, a third connective tissue layer. Given the architectural complexity of the extracellular matrix, fibroblasts are abundantly present between nerve fibers. Nerves are also highly vascular, with intrinsic vessels within the nerve trunk and a segmental nerve supply via the nerve bed [2].

When nerves are severed, such as during traumatic injury, an acute injury state is induced, characterized by biological and physical changes in the proximal and distal stumps. A key feature of the acute phase is a strong innate immune response, including an inflammatory response characterized by increased levels of pro-inflammatory cytokines (such as TNFα, IL-1α and IL-1β) and chemokines, which are quickly and locally produced at the site of injury [3,4]. In the distal stump, this up-regulation of TNFα and IL-1α drives Wallerian degeneration, a coordinated response that in mice starts 3–4 days and ends 12–14 days after injury, and that is a prerequisite for successful nerve regeneration (reviewed in [4]). Macrophages are recruited by the cytokines and chemokines, including through interleukin-17B (IL-17B) secreted by Schwann cells [5] and infiltrate the injury zone within 24 h, initially displaying a pro-inflammatory/anti-regenerative M1 identity. Resident Schwann cells clear out degraded axons and myelin, the latter of which takes up space, contains anti-regenerative molecules, and can inhibit axon renewal [3,6,7]. Within the ischemic/hypoxic microenvironment at the injury site, Schwann cells also produce exosomes that promote sustained M1 polarization [5], thereby slowing the transition to pro-regenerative M2 macrophages that support successful nerve regrowth. The early and synchronized activities of Wallerian degeneration may lead to more effective nerve repair [8,9]. Concurrently, the basal lamina compacts into Bands of Bungner, along which injured neurons regrow. Finally, resident cells up-regulate and secrete neurotrophic factors, which also help neuronal survival and regenerative axonal growth [4].

Beyond the initial (acute) injury phase, over a time frame of weeks to months, nerves that remain unrepaired progressively enter a chronic injury state. This state is characterized by numerous changes, including a change in the extracellular matrix structure of the distal stump, the down-regulation of neurotrophic factors, and changes in the recruitment, persistence, identity, and pro-regenerative activity of Schwann cells, inflammatory mediators, and immune cells. This all may be a consequence of chronic inflammation, which can have major adverse effects, locally and systemically [10]. Long-term impacts include limitations on the number of axons that can grow through a distal stump as well as degenerative changes in targeted motor and sensory organs [11,12]. In the proximal stump, a number of changes also occur, including initial recoil of the nerve stump, a disorganized cytoskeleton at axonal termini, and impaired axonal transport [13,14]. A particularly adverse response in adequately repaired or unrepaired nerves in a chronic injury state is the presence of neuromas at the injured stump, which emerge as a result of uncontrolled neuronal tissue growth at the site of nerve injury [15,16].

## 2. Scope of This Review and Methods

The literature suggests that several of the degenerative and regenerative processes summarized above may be strongly influenced by the immune response and particularly, a component of the immune response known as the complement system. The primary goals of this review are to summarize the role of the complement system in a variety of degenerative and regenerative processes following nerve injury as well as highlight knowledge gaps for further investigation towards therapeutic development. Given this scope, this review is not formally classified as a systematic review or meta-analysis.

Articles were identified in the PubMed database using various combinations of search terms including: “complement,” “peripheral nerve,” “peripheral nerve injury,” “inflammatory,” “immune,” “regeneration,” “degeneration,” “macrophage,” “neutrophil,” “B-cell,” “T-cell,” “natural killer cell,” “mast cell,” “monocyte,” “dendritic cell,” “toll-like receptor,” “spinal cord injury,” and “bone marrow”. These terms were rearranged using Boolean operators to yield a variety of papers. For example, one such search was “complement” AND “peripheral nerve injury” AND (macrophage OR monocyte) AND (inflammatory OR immune). Another was “peripheral nerve injury” OR “spinal cord injury” AND (complement) AND (“B-cell” OR “T-cell”). Duplicate articles, non-English articles, and manuscripts that had not undergone peer review at the time of the search (through October 2025) were eliminated. Reviews and articles examining complement pathways in analogous contexts other than nerve injury were included to support the posed hypotheses.

## 3. The Complement System (A General Overview)

**Figure 1:**
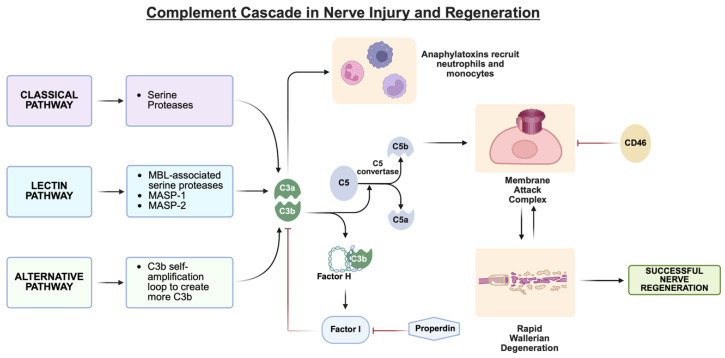
Summary of the key components of the complement cascade that influence MAC formation in the context of peripheral nerve injury, degeneration, and regeneration. The three activation pathways of the complement system converge to influence MAC formation and Wallerian degeneration. This schematic is not comprehensive and highlights the key regulatory nodes as supported by the literature; Figure 2 describes the cellular influences on these pathways in more detail. Created in BioRender. Shah, S. (2026) https://BioRender.com/3x8shvu.

The complement system, an integral component of the innate immune system, is composed of more than 40 proteins that are dissolved in the blood, body fluids and tissues. Playing a large role in tissue homeostasis as well as injury response and repair, the complement system is a rapid first-line defense against invading pathogens. Not only does it detect and send signals for the elimination of foreign debris, it also identifies unregulated or unhealthy self-cells [17]. Its main activation pathways are generally classified as the classical, alternative and lectin pathways (Figure 1). While each pathway is differentially activated, they all converge in the creation of C3a and C5a, which are pro-inflammatory mediators (anaphylatoxins), and the C5b fragment, which forms the C5b-9 membrane attack complex (MAC). The MAC causes cell lysis by punching holes in the cell membrane of invading pathogens [18].

**Figure 2:**
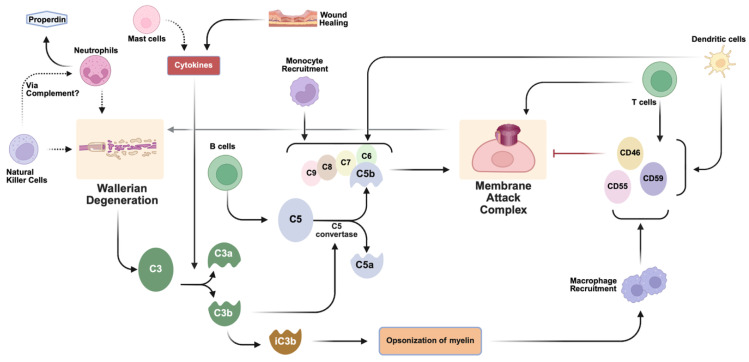
Summary of key immune cell influences on complement pathways following peripheral nerve injury. Schematic is not comprehensive and indicates cellular influences on key regulatory nodes influencing MAC formation and Wallerian degeneration as supported by the literature. Dotted lines indicated hypothesized interactions. Level of evidence for key points is summarized in Table 1. Created in BioRender. Shah, S. (2026) https://BioRender.com/tokpay1.

Although the complement system is a much-needed defense against invading pathogens, it needs to be tightly regulated as it is not foreign pathogen/tissue specific; indeed, an unchecked complement response can damage healthy host cells and tissue. One component of auto-regulation is a negative feedback loop wherein complement protein C3b binds to factor H, inducing factor I to feedback and inhibit C3b. In opposition, complement up-regulators like properdin will inhibit factor I. Another auto-regulatory mechanism is the fact that C3b molecules have short half-lives, so they can only act on their site of activation before decaying. This prevents their spread to other sites and limits the spatial extent of healthy host cell destruction [19,20,21]. Beyond defense against pathogens, a properly regulated complement system is also important for wound healing and the repair of tissues, including nerves. In the subsequent sections we will detail findings associated with the roles and regulators of complement activation in the context of peripheral nerve injury and regeneration.

## 4. Local Complement Activation During Peripheral Nerve Injury and Nerve Regeneration

Within nerves, complement mRNA is found in the peripheral nerve trunk and is most likely produced by Schwann cells. The axons of a peripheral nerve contain complement proteins; however, they do not contain any elements that regulate complement. Rather, the regulatory proteins are found in the perineurium, the connective tissue that encases and protects a nerve fascicle, as well as the myelin sheath. This suggests that the axon is at a higher risk of facing the consequences of an unchecked complement reaction due to a lack of complement-regulatory proteins [22]. Finally, MACs are believed to be formed only in the vasculature of the nerve [22]. This compartmentalization of complement participants likely influences the timing and the spatial spread of the complement response after nerve injury.

In injured nerves, complement pathways are activated and contribute to the regulation of Wallerian degeneration (Figure 1 and Figure 2). Such regulation must be tightly controlled. On one hand, unchecked or excessive clearance during Wallerian degeneration can damage nearby healthy or regrowing axons, but, in contrast, lingering degenerative material will impede the nerve’s ability to regenerate [4]. While the timing of regulation is not completely clear, it is known that complement activation and Wallerian degeneration are closely coupled following nerve injury. The primary influence on Wallerian degeneration is through MACs. Upon traumatic injury to the nerve, nerve stumps distal to the site of injury begin Wallerian degeneration. Within an hour after injury, degenerated myelin activates resident complement proteins, likely independent of circulating proteins [22]. Mechanical or biochemical disruption of axons exposes myelin and apoptotic membranes, which in turn bind C1q and activate the classical complement pathway. In parallel, the alternative pathway auto-activates on injured Schwann cell surfaces, generating rapid cleavage of C3 and C5 and producing the inflammatory mediators C3a, C3b, C5a, and C5b [23,24]. These complement proteins create MACs that emerge within 12 h and remain up to 3 days. MACs accelerate Wallerian degeneration by destroying the axons and myelin nearby [25]; without MACs, Wallerian degeneration, which typically will have been initiated by 3 days post-injury, would occur too slowly, impeding nerve regeneration [4,26]. Degenerated myelin also induces the complement system to create C3bi, a complement protein that opsonizes myelin and targets it for removal. Complement C3 can then bind to opsonized myelin, causing a 2- to 3-fold increase in myelin phagocytosis and subsequent Schwann cell migration and remyelination [27,28]. If the destroyed myelin is not removed fast enough, it will impair nerve regeneration. Therefore, the complement system accelerates the recruitment of professional phagocytes to the injury site [29].

Interactions between complements and Wallerian degeneration also affect axonal regeneration through the regulation of neurotrophic factors (described generally here, and in more detail in subsequent sections). For example, *Wld^s^* (Wallerian degeneration slow) mice—which via a spontaneous autosomal-dominant 85 kb tandem triplication on chromosome 4 exhibit slow and delayed Wallerian degeneration—show suppressed up-regulation of neurotrophic factors after peripheral nerve injury [30,31]. Complement cascades have not been studied in *Wld^s^* mice; however, given the critical role of MAC in modulating rapid Wallerian degeneration [26] and the complement’s contribution to creating the MAC, the lack of neurotrophic up-regulation in *Wld^s^* mice might link the complement system and neurotrophic factor expression. For example, the C5a-like receptor 2 (C5aR2/C5L2) has been shown to negatively modulate brain-derived neurotrophic factor (BDNF) secretion in dental pulp stem cells [32]. Given that BDNF enhances nerve regeneration [33], such a neurotrophic factor may connect complement and nerve regeneration. In addition, the complement system interacts with inflammasomes, which play a role in the inflammatory response in tissue and nerve injury and are usually present at the site of injury [34,35,36]. Inflammasomes are activated and promote the release of interleukin-1β (IL-1β) and IL-18. These inflammatory cytokines contribute to regenerative processes in both the proximal and distal stumps.

## 5. Cellular Interactions with the Complement System in Peripheral Nerve Injury

**Figure 3:**
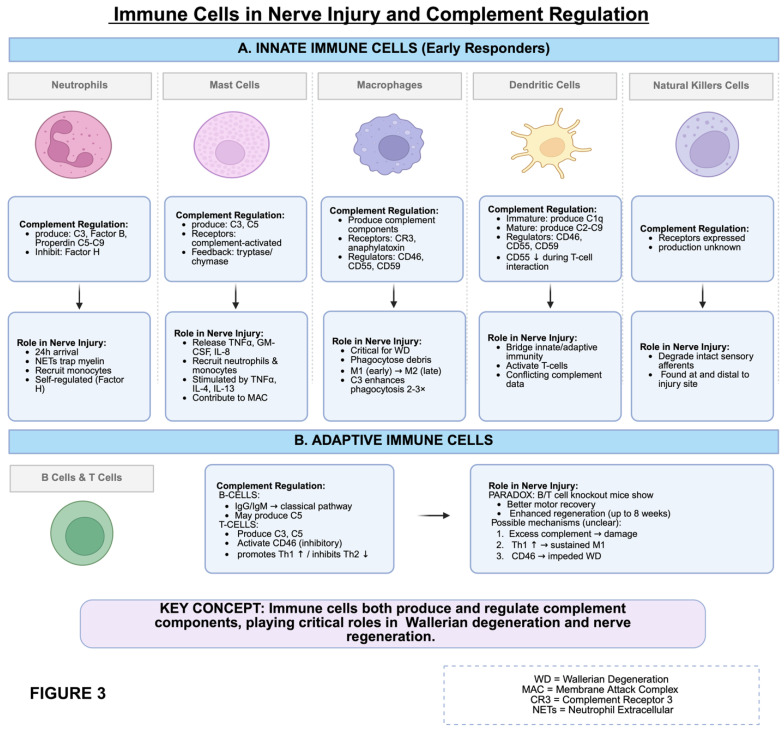
Additional details related to the influence of innate and adaptive immune cells on complement regulation as related to nerve degenerative and regenerative pathways. The level of evidence for the key points is summarized in Table 1. Arrows represent a conceptual influence of complement regulation on a particular role or roles in the response to nerve injury. Created in BioRender. Shah, S. (2026) https://BioRender.com/i8paj8h.

The complement system must closely self-regulate in order to limit unnecessary damage to intact nerves. Immune cells (both innate and adaptive), cytokines, growth factors, and factors H and I are all components that can directly or indirectly result from complement activation but can feed back to regulate it as well. The following sections discuss the complement system’s interaction with a variety of inflammatory and immune cells and their associated regulatory pathways in the context of nerve injury (Figure 2 and Figure 3). While several complement-associated pathways have been directly linked to the neurodegenerative response in models of nerve injury, we have hypothesized others based on known immunologic roles for complements (Table 1).

### 5.1. Neutrophils and Monocytes/Macrophages

While the interplay between the complement system and neutrophils has not been studied in the context of peripheral nerve injury, our general understanding of neutrophil interactions with complements provides some clues as to their relevance. The complement system can recruit neutrophils and in turn, neutrophils can create and regulate complement components [37]. For example, neutrophils contain complement factors like C3, factor B and properdin [38]. Factor C3 is located on neutrophil membranes and downregulates the complement system by binding to factor H, which inhibits the alternative pathway. On the other hand, neutrophils can up-regulate the complement system by producing properdin, a positive regulator of the complement system’s alternative pathway which stabilizes the C3b/Bb convertase enzyme complex [38,39,40,41]. The mechanisms underlying a neutrophil’s decision to create C3 versus properdin are not well known, but there is assumed to be a balance between the creation of the two. It is speculated that the MAC may destroy neutrophils under certain conditions [42], so neutrophils may create factor H to avoid this fate.

Neutrophils are early arrivals, via circulation, to the scene of a peripheral nerve injury, helping macrophages phagocytose degraded axons and myelin [43]. When neutrophils arrive at the injury site, they recruit circulating monocytes that (1) differentiate into macrophages in the nerve for increased debris clearance and (2) form neutrophil extracellular trap (NET) structures that trap and break down myelin debris [44]. Early studies supported a role for neutrophils in creating C5, C6, C7, C8 and C9 [45], which are needed to create MACs, thus implying that neutrophils could influence Wallerian degeneration via complements [43]. However, more recent work in spinal cord injury models noted the absence of C5–C9 transcripts, suggesting alternative cellular influences on complement protein production [46]. In either scenario, up-regulation of complement proteins is likely to be transient, given the inhibition of complements by neutrophils via C3/factor H noted above, to reduce the likelihood of complement-induced damage.

The fact that the recruited monocytes are so integrated with the complement system suggests an additional indirect relationship between neutrophils and the complement system in peripheral nerve injury repair [38,47,48]. This is especially evident as the tissue-specific form of monocytes and macrophages can regulate the complement system through the creation of additional complement components and regulators, such as CD46 (a co-factor for the cleavage of C4b and C3b [49]), CD55 (which hastens the dissociation of C3 and C5 convertases that form on macrophage surfaces [50]) and CD59 (which prevents MAC formation by inhibiting C8–C9-associated activities [51]). Macrophage activity in degenerating nerves is dependent on C3, as confirmed in nerves injured in the presence of an anti-C3 antibody [27]. Macrophages also contain complement receptors on their surface, including anaphylatoxin receptors that bind C3a and C5a, further linking macrophage activity to the inflammatory response. Collectively, these data suggest that the recruitment of monocytes to the site of an injured nerve, and their subsequent differentiation into macrophages, can have complement-related impacts on peripheral nerve injury. This continued complement activation may sustain pro-inflammatory M1 macrophage polarization and inhibit the shift to pro-regenerative M2 macrophages. Removing this inhibition has been proposed by inhibiting complements [52] or the usage of maresin 1 to promote M2 polarization [53] (the former in a nerve injury model and the latter in a lung injury model).

### 5.2. Mast Cells

Mast cells typically play a role in allergic and inflammatory responses of the innate immune system [54]. Mast cells can also create complement proteins (C3 and C5) and contain complement protein receptors on their surface which allow for complement proteins to activate mast cells [48,54,55,56]. After peripheral nerve injury, within the first week, mast cells increase at the injury site. These cells release biogenic amines such as histamine and heparin as well as neurotrophic factors such as nerve growth factor (NGF) that support neuronal survival. These mediators increase vascular permeability, providing access to the injury site for circulating cells [57]. In a later phase, mast cells release tryptase and chymase and cause degranulation. These serine proteases reciprocally activate complement proteins, therefore contributing to a positive feedback loop [54]. It was additionally found that the secretion of complement proteins (i.e., C3) from mast cells is up-regulated in response to the cytokines TNFα and IL-4 or IL-13 [54,55]. In nerves, mast cells release pro-inflammatory cytokines (TNFα, GM-CSF, and IL-8) and chemokines (CCL2, CCL3 and CCL4) [58]. Granulocyte–macrophage colony-stimulating factor (GM-CSF) initiates the differentiation and proliferation of monocytes, activation of macrophages, and recruitment of neutrophils [59]. IL-8 is commonly known to promote chemotaxis in neutrophils and stimulate phagocytosis upon neutrophils’ arrival to the target, while CCL2 and CCL3 are involved in recruiting monocytes [58]. The release of cytokines and chemokines from mast cells is suggestive of a relationship between the complement system, mast cells, and cytokines/chemokines during peripheral nerve injury. Given that effective Wallerian degeneration depends on the release of cytokines (like TNF-α) and the formation of an MAC (of which C5 is a critical component), one might predict that during peripheral nerve injury, mast cells release cytokines, including (but not limited to) TNF-α. In turn, cytokines may stimulate mast cells to release complement proteins, such as C5, which may help create the MACs needed for Wallerian degeneration. Although none of these hypotheses have been tested, the above concepts represent a starting point to connect the roles of mast cells, the complement system, and Wallerian degeneration in nerve injury.

### 5.3. B-Cells and T-Cells

B-cells and T-cells are components of adaptive immunity but also interface with complement pathways. B-cells secrete the antibodies IgG and IgM, which can activate the classical pathway of the complement system. There are also some complement receptors on B-cells, and B-cells produce the complement components C3 and factor I, which participate in autocrine signaling pathways [48,60,61]. A recent study shows that activated B-cells, for their own maturation, down-regulate the complement regulator DAF/CD55 on their own surfaces to permit local C3a and C5a generation [62]. T-cells, on the other hand, can produce complement factors and also regulate the complement system by activating CD46, a complement regulator [63].

To discover the role of B- and T-cells in peripheral nerve injury, a mouse mutant was examined that did not contain T- and B-cells [64]. Interestingly, following femoral nerve injury, these mutant models had better motor recovery and nerve regeneration up to eight weeks after the injury occurred. The authors’ hypothesis was that differential macrophage activation in the mutant mice allowed for better regeneration. As cytokines secreted by B- and T-cells target macrophages, the authors hypothesized that the lack of this lymphocyte-specific cytokine secretion may positively affect macrophage roles in the regenerative process [64].

While the role of the complement system was not considered in this study, these findings may be interpreted in the context of complement system function. One possible explanation is that the antibodies released from B-cells induce the complement system unchecked, which may be caused by B-cells’ down-regulation of complement inhibitor DAF/CD55 [62]; too much complement activation can cause more damage than is needed, therefore hindering regeneration. Additionally, it has been found that the complement system activates Th1 production but inhibits Th2 production [63,65]. Generally, Th1 cells produce cytokines that cause a pro-inflammatory response, which must be balanced by anti-inflammatory cytokines produced by Th2 cells [66]. Over-activation of the complement system may increase Th1 cytokines, leading to an increased pro-inflammatory response and impeding nerve regeneration. In contrast, without B- and T-cells, reduced complement activity could enhance nerve regeneration.

Another plausible explanation is related to the T-cell activation of CD46, a complement regulator. Over-activation of CD46 may inhibit the complement system, impeding Wallerian degeneration and therefore impairing regeneration. However, this scenario may be less likely, considering that T-cells also produce several different complement proteins, including C3 and C5 which are needed for the MAC. While it is less-known to what extent B-cells can produce complement proteins, there is some evidence indicating that they create and secrete C5 as well as other complement proteins [48]. This would suggest that B- and T-cells in fact aid in regeneration, in paradoxical contrast to the previous study. To better frame the study’s conclusions through the lens of the complement system, we need to investigate what aspects of complement regulation (e.g., positive/negative, as well as timing) are more directly or sensitively influenced by B- and T-cells.

### 5.4. Dendritic Cells

Dendritic cells, which infiltrate the injured sciatic nerve and ipsilateral dorsal root ganglia alongside neutrophils, macrophages, and lymphocytes [67], bridge innate and adaptive immunity and act as antigen-presenting cells, which phagocytose and present an antigen to cells of the adaptive immune system. Dendritic cells are immature until they receive maturation signals, at which point they can then activate T-cells. In their immature state they can produce the complement protein C1q, although there is debate on whether they continue to produce this protein once mature [48,68]. Mature dendritic cells produce many complement proteins such as C2–C9 [48], as well as the complement regulators CD46, CD55 and CD59 [69,70].

CD 55 inhibits all three pathways (classical, alternative and lectin) of the complement system, while C1q is part of the classical system. The possibility that C1q is unable to be created in a mature dendritic cell, whose job is to activate T-cells, might suggest that parts of the complement system cannot work in tandem with adaptive immunity (or at least, need to be turned off to activate T-cells). However, CD55 is down-regulated during the interaction between dendritic cells and T-cells [71], suggesting that complement activation is important for this interaction. These are apparently conflicting results, which highlight the importance of both complement activation and inhibition during T-cell and dendritic interaction.

### 5.5. Natural Killer Cells

Part of the innate immune system, natural killer (NK) cells are cytotoxic lymphocytes known to contain many complement receptors on their surfaces, allowing them to interact with complement factors [48,72,73]. In a tumor immunity scenario, natural killer cells express CR3, which suppresses natural killer cell cytotoxicity [74]. However, it is not known whether they can produce and release complement factors themselves. In injured nerves, natural killer cells are found at and distal to the injury site and trigger the degradation of axons in the PNS [75]. The specific relationship between natural killer cells and the complement system during peripheral nerve injury is unclear, but it can be assumed that there is some interaction. The complement receptors, such as CR3, located on NK cells’ surface could allow for communication with the complement system during Wallerian degeneration and the clearance of damaged axons/myelin. But whether CR3 similarly suppresses natural killer cells during peripheral nerve injury remains unknown.

### 5.6. Toll-like Receptors

Toll-like receptors (TLRs) are pattern-recognition receptors expressed by a number of cell types, including immune cells and cells of the nervous system, that play a large role as infection sensors [76,77,78]. TLRs are quickly activated and are closely intertwined with the complement system. Both TLRs and complements can be activated by the same microbial molecules and they can regulate each other through the interaction of their various receptors [77,79,80]. For example, the expression of complement components is up-regulated when TLR4 is activated. Additionally, complements can inhibit TLR production of different cytokines, including the IL-12 family [80,81,82], which causes the differentiation of Th1 from naive CD4+ T-cells [83]. Therefore, though the complement system does increase levels of Th1 [41,43], it may auto-regulate by down-regulating Th1 development via TLR pathways. Additionally, TLR2 can activate complement receptor 3 (CR3), which, when on the surface of macrophages, further up-regulates TLR signaling [77,84].

The crosstalk between TLRs and complements is an important aspect of nerve injury and regeneration. Concurrent to C3 deposition, TLR1, TLR2, TLR3, TLR4, and TLR7 are highly activated in peripheral nerves after axotomy, nerve crush, or ligation and can help activate Schwann cells [85,86,87,88]. In a positive feedback loop, the activation of Schwann cells also enhances TLR expression. The expression of TLRs in Schwann cells causes higher expression of pro-inflammatory mediators that aid in Wallerian degeneration [89]. For example, the TLR2, TLR4, TLR6, and TLR9 ligands elevate plasma IL-6, TNF-α, and IL-1β, through pathways mediated by the anaphylatoxin receptors C5aR and C3aR. This interaction appears to amplify inflammation through enhanced NF-κB and MAPK activation [90]. Additionally, after injury, macrophage recruitment to the sciatic nerve distal stump is significantly less in TLR-deficient mice than in wild-type mice. As a consequence, TLR-deficient mice had less effective myelin clearance, Wallerian degeneration and axonal regeneration [85,89]. TLRs also locally increase neurotrophin production, additionally enhancing regeneration [89,91]. However, TLR activation must be controlled, as excessive activation may contribute to chronic neuropathic pain [92].

## 6. Parallels to Complement Activation in Wound Healing and Spinal Cord Injury

### 6.1. Coagulation Factors and Wound Healing

Wound healing in the skin is well-studied and may have several similarities to pathways following nerve injury that influence nerve regeneration. Although coagulation pathways have not been studied in nerve injury, vascular and nerve injury usually occur concurrently and therefore likely trigger the activity of coagulation and wound healing factors.

Platelets, a major player in hemostasis, have been implicated in the activation of the complement system [37,93]. Additionally, thrombin and plasmin, involved in coagulation and fibrinolysis, respectively, activate C3 and C5 [93,94,95]. It was found that C3 and C5 help with wound healing, both accelerating the process and increasing the strength of the repair [95,96,97]. Experimentally, increasing C5 in skin wounds led to increased influx of inflammatory cells and fibroblasts as well as greater deposition of collagen. When collagen is exposed within a wound, the coagulation (clotting) cascade and platelets are activated in wound healing pathways [98].

However, plasmin can also play a regulatory role and destroy parts of the complement system needed to create the MAC [37,99]. The complement system in turn regulates parts of the coagulation cascade such as fibrin formation. Activated platelets secrete several cytokines like TNF-alpha, PDGF, TGF-beta, epidermal growth factor (EGF), fibrinogen, histamines, platelet-derived growth factor (PDGF), fibronectins and more. These all help with clotting as well as recruiting macrophages and fibroblasts to the wound [100,101].

The complement cascade in wound repair is further activated by platelet degranulation and proceeds to help recruit inflammatory leukocytes to the injury and lyse bacteria, if present [98]. After platelet and complement activation neutrophils are recruited, becoming the first inflammatory cells arriving at the wound. Neutrophils will bind to P-selectins, which are adhesion molecules on endothelial cell surfaces, and clear debris, working with the complement system to lyse bacteria [37,98]. Monocytes (which convert to macrophages on site) and lymphocytes are also recruited to the wound. In addition to phagocytosing debris, macrophages release cytokines, interleukins, transforming growth factor (TGF), platelet-derived growth factor, and tumor necrosis factor (TNF) among other factors. These activate fibroblasts and aid in angiogenesis. Wound healing also consists of the fibroplasia phase, where fibroblasts enter the wound site to help stimulate capillary growth [37,98,102,103].

In comparing wound healing and the processes after nerve injury, both include the release of cytokines that signal for TNF-alpha and IL-1 [4,100,101], two essential inflammatory regulators. Neutrophils are also some of the first inflammatory cells at the site of injury for both processes [37,43]. Therefore, both have an inflammation phase where immune cells are recruited to the injury site. The fibroplasia phase in wound healing also exists in nerve regeneration, as fibroblasts in the epineurium and perineurium help stabilize the strength of the nerve cell by synthesizing collagen (or, when misregulated, create the components of scar) (reviewed in [2,98]). Since exposed collagen triggers the coagulation cascade [98], this further ties concepts in wound healing and nerve repair together. The fact that wound healing and nerve repair share common major players indicates that these processes are likely meant to work side-by-side, with products from one process regulating aspects of the other and vice versa.

### 6.2. Spinal Cord Injury

Many aspects of the complement response after peripheral nerve injury parallel those observed after spinal cord injury (SCI). Early classical and alternative pathway activation after peripheral nerve injury is remarkably similar to the complement response described in SCI, where complement activation is a dominant feature of acute secondary injury [104,105]. In both CNS and PNS tissues, C1q binding to damaged myelin initiates opsonization, while deposition of C3b and iC3b labels debris for phagocytosis. The anaphylatoxins C3a and C5a then recruit macrophages and neutrophils which amplify local inflammation [105,106]. Both SCI and PNI also exhibit pathological consequences of sustained C5aR1 signaling. In SCI, C5aR1 activation drives neuronal apoptosis, cytokine release, and secondary lesion expansion, and genetic or pharmacological C5aR1 inhibition markedly improves outcomes [105,107]. Recent work now demonstrates that Schwann cell C5aR1 also promotes NLRP1/IL-1β inflammasome activation, pro-inflammatory cytokine production, and maladaptive macrophage recruitment after PNI [108], indicating that excessive C5aR1 activity is common across central and peripheral nerve injuries. Although direct tests in traumatic nerve injury remain limited, the available evidence from SCIs suggests that resolution of complement signaling is necessary for the transition from pro-inflammatory to pro-repair macrophage states.

Complement activation in PNI, though initiated in proximity to the injury site [22], may also reflect systemic influences. After injury, monocytes infiltrate the distal nerve in large numbers [109] and are posited to emerge from bone marrow, likely amplifying complement-mediated inflammation. Conversely, complement fragments generated at the injured nerve, particularly C5a, may circulate to the bone marrow and modulate stromal and myeloid cells, potentially influencing monocyte production and maintaining a pro-inflammatory state [110]. Additionally, nerve damage itself can exert systemic effects; sympathetic innervation of the bone marrow regulates hematopoietic stem and progenitor cell activity, and loss of this signaling after chemotherapy-induced injury impaired hematopoietic recovery [111]. This dual local–systemic response parallels SCI, where infiltrating monocytes are known to drive complement-dependent secondary injury [105], and complement dysregulation alters systemic immunity [104]. Similar systemic feedback loops may occur in PNI, contributing to chronic inflammation and impaired long-term recovery. Such possibilities, including a careful evaluation of complement-related alterations to the transcriptomes and proteomes of bone marrow progenitors, remain interesting areas of future study.

## 7. Therapeutic Implications

### 7.1. Complement as a Therapeutic Target

The above review demonstrates a number of direct and indirect influences of the complement system on nerve degeneration and regeneration. Though several of the hypothesized impacts rely on extrapolation from non-nerve literature, both within and beyond the nervous system, these studies suggest that manipulation of the complement system has important translational implications for enhancing nerve repair or preventing/overcoming barriers to nerve repair. Such approaches have not been formally introduced into the clinic. However, several preclinical pharmacological and genetic therapeutic strategies demonstrate both the potential feasibility of such approaches as well as some of the limitations and points of caution in clinical translation.

Therapeutic studies in SCI and traumatic brain injury (TBI) highlight the potential value of targeting complement signaling in PNI, considering the above-noted similarities in the complement response to central and peripheral nervous system injury. Pharmacological inhibition of C5aR1 reduced inflammation, apoptosis, and glial scarring in a mouse model of SCI [105]. Similarly, broad spectrum complement inhibition using Vaccinia Virus Complement Control Protein (VCP) protects neural tissue and improves structural outcomes in rat SCI models [112,113]. Complement inhibition has also improved outcomes in TBI by reducing secondary neuroinflammation and preventing complement-mediated tissue damage [107]. Although these C5aR1 antagonists and VCP have demonstrated therapeutic benefit by reducing inflammation, apoptosis, complement deposition, and improving tissue preservation [105,107,112,113], direct tests of these strategies in traumatic PNI have not been performed. Thus, the utility of such therapies remains a biologically supported inference rather than a tested treatment.

Another promising modulator of the complement system is recombinant human c1 esterase inhibitor (rhC1INH), which inhibits the classical complement pathway via the C1 protein. This therapy (e.g., Ruconest/Conestat alfa) has been deployed clinically for a number of indications, including angioedema and chronic kidney disease [114,115]. Compellingly, treatment of rats with rhC1INH reduces acute damage in the injured nerve, though the long-term effects of rhC1INH on nerve regeneration outcomes as well as any systemic effects are unknown [26].

While modulation of complements may be powerful, therapeutic interventions should not completely inhibit the complement system in the context of nerve regeneration. Indeed, complete knockout of the complement system results in fewer regenerating axons and delayed axonal regeneration than controls after sciatic crush injury in rats [23]. Additionally, rats with a depleted complement system had fewer macrophages recruited and activated. Macrophages are needed for axonal regeneration, as they clear cellular debris and promote the creation of trophic and tropic factors aiding in axonal growth [23]. Therefore, clinical interventions need to be done on select components of the complement system rather than knocking it out entirely.

### 7.2. Therapeutic Windows and Delivery

Several animal models and clinical studies in peripheral nerve injury models and other injury and disease states suggest a therapeutic window ranging from a few hours to one week for effective complement-related interventions. Treatment with soluble complement receptor 1 (sCR1) in a rat sciatic nerve crush injury model blocked both systemic and local complement activation when administered after injury. This resulted in protection of the nerve from axonal and myelin breakdown at 3 days post-injury and strongly reduced macrophage infiltration and activation [116]. Similarly, C1 inhibitor (Cetor) administered 3 days after injury resulted in diminished MAC deposition and myelin breakdown inhibition and decreased macrophage infiltration, though early signs of axonal degradation were still visible compared to sCR1 treatment [116]. The efficacy of this timing is consistent with the timeline of Wallerian degeneration via MAC deposition, which occurs by 3 days post-injury in untreated animals [26]. In neuropathic pain models using spinal nerve ligation, daily systemic injections of sCR1 up to 4 days post-injury markedly suppressed C3 deposition and immune cell recruitment, while producing significant alleviation of thermal hyperalgesia and mechanical allodynia for up to 7 days. And, in an experimental stroke model, recombinant human C1-INH administered up to 18 h after transient ischemia and 6 h after permanent ischemia resulted in markedly reduced cerebral damage [117,118]. Analogously, in ischemic stroke models, both C1-INH and intravenous immunoglobulin significantly reduced brain infarction, neurological deficits, and mortality when administered up to 6 h after reperfusion onset [118,119]. While early intervention does appear to support more robust protection against axonal damage and an excessive immune response, the current data remain limited and are primarily derived from small patient cohorts or animal studies [116,119,120]. In addition, very little is known about the long-term efficacy of complement therapies in suppressing or delaying the transition from an acute nerve injury to a chronic nerve injury state, or whether prolonged inhibition of complement components may perturb the longer-term dynamics of complements required for functional regeneration. Thus, further research is needed to establish precise therapeutic windows and personalized regimens for complement inhibition, including attention to specific mechanisms of action, the duration of therapy, and the appropriate evaluation timepoints.

Strategies for therapeutic delivery, then, follow insight into therapeutic windows. The above studies relied primarily on systemic delivery of complement-related therapeutics and restricted their evaluation primarily to nerve structure and function within several days of injury. However, a more thoughtful therapeutic solution would integrate the manipulation the local neurodegenerative and regenerative response (free from systemic effects), and account for the fact that nerve regeneration occurs over an extended time course and requires temporally controlled modulation of the local microenvironment to support axonal elongation, myelination, and functional reinnervation. Short-lived bolus drug exposures resulting from systemic pharmacologic therapies are poorly matched to this intent, as they cannot reliably maintain therapeutic concentrations at the nerve repair site. Nerve-interface and nerve-wrap devices designed to act as localized drug reservoirs provide a compelling solution by physically constraining pharmacological agents at the site of injury while enabling controlled, prolonged release aligned with the regenerative timeline. There is a precedent for such efforts. Drug-eluting nerve wraps delivering tacrolimus (FK506) have demonstrated enhanced axonal regeneration and accelerated functional recovery through sustained local exposure with minimal systemic drug levels [121,122]. Similarly, nerve conduits incorporating neurotrophic factors such as NGF or GDNF via microsphere-based or scaffold-integrated delivery systems have shown that spatiotemporally controlled release over several weeks can support neurite outgrowth, Schwann cell migration, and improved histological and functional outcomes [123,124]. Together, these data support the rationale for tunable engineered devices that combine regenerative guidance with sustained local pharmacologic delivery, capable of addressing the prolonged and dynamic biology of complements in the setting of peripheral nerve repair.

### 7.3. Complementary Therapeutic Targets and Strategies

While this review has largely characterized the MAC as a key node in the complement regulation of nerve regeneration, we acknowledge that there is some controversy surrounding the extent to which MACs influence nerve regeneration. For example, MAC residue on nervous tissue can cause excessive calcium influx, which activates calpains, causing structural damage to the nerve [26]. In fact, calpain inhibitors have been found to prevent axonal cytoskeletal loss in models of complement-mediated CNS injury [26], suggesting their potential as a supplementary treatment strategy in the PNS as well.

We largely constrained discussion of complements to the acute response to injury. However, the fact that chronic injury results in the down-regulation of neurotrophic factors and reduced trophic support from Schwann cells—two processes that interface with the complement system—suggests that the complement system is also likely to influence chronic nerve injury. As noted above, the transition from acute to chronic injury has been poorly studied. It is possible that the impact of inadequate nerve repair or a “chronic-like” state may be seen in neuromas, which are the result of uncontrolled neuronal tissue growth at the site of nerve injury [15]. In addition to Schwann cells and fibroblasts, neuromas contain bundles of axons that are improperly woven together [16]. Neuromas cause neuropathic pain due to increased sensitivity and are hard to manage [16,125,126]. Possible factors in neuroma formation are the pro-inflammatory cytokines TNF-α, IL-1 β and IL-6, which are up-regulated in rats who underwent sciatic nerve transection that resulted in neuromas versus those without neuromas [15], as well as TLRs [127]. As such cytokines stimulate mast cells to release complement proteins that then form the MAC to kickstart Wallerian degeneration, we speculate that neuroma formation and progression may be influenced by complement activation. This hypothesis remains to be tested. Independent of neuroma formation, B-cells may also drive mechanical allodynia after nerve injury through IgG-mediated complement activation [128].

### 7.4. Other Translational Considerations

It should be noted that many preclinical studies have been performed in rodent models of nerve injury [129]. However, the timing of complement system activation may differ in mice and rats versus other mammals, including primates. For example, axonal degeneration was detected as early as 36 to 44 h in mice and rats, but after one week in baboons [4,130,131]. Thus, these temporal factors need to be included in dosing strategies. In addition, there are several practical factors that affect such therapeutic strategies, including size of animals and differences in metabolism that may influence biodistribution [129,132,133].

Differences in gene regulation between humans and mice poses another challenge in using mice as a model for the complement system. For example, in humans, the complement system is partially controlled by genes within the Regulators of Complement Activation (RCA) cluster on chromosome 1. While mice also have the RCA cluster, they are missing the locus for Complement Receptor 1 (CR1/CD35) [134]. CR1/CD35 acts as a receptor for and a negative regulator of the complement system. Despite the creation of mouse models with a modified CR1 to be more human-like, the nuances in complement cascade regulation are missed [135].

Finally, within humans too there is variability in relation to complement activity. For example, single nucleotide polymorphisms (SNPs) in C3 and C5, or factor H, have been identified and are associated with loss of function; this can increase the risk of patients for infections or a subset of chronic diseases (like SLE) [136]. These variants may even modulate the therapeutic response, thereby additionally affecting treatment outcomes. Thus, personalized treatment may be enhanced based on the genetic determinants of complement function.

## 8. Conclusions

There has been a considerable amount of research performed on inflammatory influences on nerve regeneration. However, far less has been studied about the complement system and how this versatile and important regulator intersects/interacts with inflammatory, immune, and regenerative processes. These remain exciting areas of future research and possible avenues for developing therapeutic interventions.

## Figures and Tables

**Table 1:** Immune cell interactions with the complement system in the context of peripheral nerve injury. The evidence level for the conclusions drawn is provided in italics in the right-most column.

Immune Cell Type	Known Interaction with the Complement System and/or Role in PNI	Conclusions (*Evidence Level*)
**Neutrophils**	Neutrophils serve as regulators of the complement system. Factor C3, located on neutrophil membranes, down-regulates the complement system, whereas properdin, produced by neutrophils, prolongs the half-life of C3 convertase, stabilizing the complement system. Upon arrival at the PNI site, neutrophils aid in Wallerian degeneration through the phagocytic clearance of myelin debris and formation of neutrophil extracellular traps (NETs).	Neutrophils and the complement system have parallel roles in Wallerian degeneration. *Neutrophils and the complement system’s parallel roles have been shown in peripheral nerve injury models; however, no direct mechanistic interaction between the two has been evidenced in these models.*
**Macrophages**	Macrophages create and express CD46, CD55 and CD59, all regulators of the complement system. They also contain anaphylatoxin receptors on their surface, which bind C3a and C5a and trigger immune cell activation, chemotaxis and inflammatory mediatory production.	Complement proteins (specifically C3) are necessary for macrophage invasion of degenerating nerves. *Peripheral nerve injury model.*
		Complement activation in peripheral nerve injury may sustain pro-inflammatory M1 macrophage polarization and inhibit the shift to pro-regenerative M2 macrophages. Removing this inhibition has been proposed by inhibiting complement or usage of maresin 1 to promote M2 polarization. *Hypothesized based on general immunology.*
**Mast Cells**	Mast cells create complement proteins C3 and C5 and contain receptors on their surface which allow for complement proteins to activate them. Mast cells release pro-inflammatory cytokines and serine proteases at peripheral nerve injury sites. Secretion and activation of complement proteins is up-regulated by cytokines and serine proteases, respectively.	Mast cells release cytokines, including (but not limited to) TNF-α. In turn, cytokines may stimulate mast cells to release complement proteins, such as C5, which may help create MACs needed for Wallerian degeneration. *Hypothesized based on general immunology.*
**B-cells**	B-cells produce complement components C3 and factor I which participate in autocrine signaling pathways important for B-cell functions. B-cells also, for their own maturation, down-regulate the complement regulator DAF/CD55 on their own surfaces to permit local C3a and C5a generation. B-cells secrete the antibodies IgG and IgM, which can activate the classical pathway of the complement system. In a mutant mouse model without B-cells, there was better motor recovery and nerve regeneration 8 weeks post-injury.	The lack of lymphocyte-specific cytokine secretion positively affects macrophages’ role in regenerative processes. *Hypothesized based on general immunology.*
		Antibodies released from B-cells induce the complement system unchecked, which may be caused by B-cells’ down-regulation of complement inhibitor DAF/CD55; too much complement activation can cause more damage than is needed, therefore hindering regeneration. *Hypothesized based on general immunology.*
		B-cells drive mechanical allodynia after PNI through IgG-mediated complement activation. *Peripheral nerve injury model.*
**T-cells**	T-cells can produce complement factors and regulate the complement system by activating CD46. The complement system activates Th1 production but inhibits Th2 production. Th1 cells produce cytokines that cause a pro-inflammatory response, which must be balanced by anti-inflammatory cytokines produced by Th2 cells.	Over-activation of CD46 may inhibit the complement system, impeding Wallerian degeneration and therefore impairing regeneration. However, this hypothesis is less likely. *Hypothesized based on general immunology.*
		Over-activation of the complement system may increase Th1 cytokines, leading to an increased pro-inflammatory response and impeding nerve regeneration. *Hypothesized based on general immunology.*
**Dendritic Cells**	Mature dendritic cells produce many complement proteins such as C2-C9 as well as the complement regulators CD46, CD55 and CD59, but do not create C1q, which is required to activate T-cells. Following PNI, dendritic cells infiltrate the injured sciatic nerve and ipsilateral dorsal root ganglia alongside neutrophils, macrophages, and lymphocytes.	Dendritic cell interactions with T-cells via complement proteins are in balance between a less activated state (via C1q) and an activated state (CD55 down-regulation), suggesting that complement pathways are important for this interaction. *Hypothesized based on general immunology.*
**Natural Killer Cells**	Natural killer cells contain many complement receptors on their surface, including CR3. In injured nerves, natural killer cells are found at and distal to the injury site and trigger the degradation of axons in the PNS. Thus, there is likely interaction between natural killer cells and the complement system in peripheral nerve injury.	NK cells are present in degenerating nerves. *Peripheral nerve injury models.*
		The complement receptors such as CR3 located on NK cells’ surface could allow for communication with the complement system during Wallerian degeneration and the clearance of damaged axons/myelin. *Hypothesized based on general immunology.*
**Toll-Like Receptors**	Both TLR and complement signaling are activated following peripheral nerve injury and interact to amplify inflammatory responses. In sciatic nerve crush and spinal nerve ligation models, TLR2 and TLR4 are up-regulated on Schwann cells and glial cells, which triggers the production of pro-inflammatory mediators that recruit immune cells to the injury site. In parallel to this, C3 is deposited in injured nerves from 6 h to 7 days after injury. TLR2- and TLR4-deficient mice show impaired macrophage recruitment, delayed Wallerian degeneration, and compromised functional recovery after sciatic nerve lesion. Similarly, complement depletion using cobra venom factor reduces macrophage infiltration and activation, delays myelin clearance, and impairs axonal regeneration.	TLR ligands cause complement-dependent elevation of plasma IL-6, TNF-α, and IL-1β, with this regulatory effect mediated by the anaphylatoxin receptors C5aR and C3aR. This interaction appears to amplify inflammation through enhanced NF-κB and MAPK activation. *Peripheral nerve injury models.*
		There is molecular crosstalk allowing for appropriate macrophage recruitment and Wallerian degeneration between TLRs and the complement system in peripheral nerve injury. *Peripheral nerve injury models.*

## Data Availability

Not applicable.
